# Does Upper Cervical Manual Therapy Provide Additional Benefit in Disability and Mobility over a Physiotherapy Primary Care Program for Chronic Cervicalgia? A Randomized Controlled Trial

**DOI:** 10.3390/ijerph17228334

**Published:** 2020-11-11

**Authors:** Vanessa González-Rueda, César Hidalgo-García, Jacobo Rodríguez-Sanz, Elena Bueno-Gracia, Albert Pérez-Bellmunt, Pere Ramón Rodríguez-Rubio, Carlos López-de-Celis

**Affiliations:** 1 Faculty of Medicine and Health Sciences, Universitat Internacional de Catalunya, 08195 Barcelona, Spain; vgonzalez@uic.es (V.G.-R.); jrodriguezs@uic.es (J.R.-S.); aperez@uic.es (A.P.-B.); prodriguez@uic.es (P.R.R.-R.); carlesldc@uic.es (C.L.-d.-C.); 2 Fundació Institut Universitari per a la recerca a l’Atenció Primaria de Salut Jordi Gol i Gurina, 08007 Barcelona, Spain; 3 Facultad de Ciencias de la Salud, Unidad de Investigación en Fisioterapia, Universidad de Zaragoza, 50009 Zaragoza, Spain; ebueno@unizar.es

**Keywords:** craniocervical, neck pain, physical therapy, spinal manipulation, range of motion

## Abstract

*Introduction*: Neck pain is a condition with a high incidence in primary care. Patients with chronic neck pain often experience reduction in neck mobility. However, no study to date has investigated the effects of manual mobilization of the upper cervical spine in patients with chronic mechanical neck pain and restricted upper cervical rotation. *Objective*: To evaluate the effect of adding an upper cervical translatoric mobilization or an inhibitory suboccipital technique to a conventional physical therapy protocol in patients with chronic neck pain test on disability and cervical range of motion. *Design*: Randomized controlled trial. *Methods*: Seventy-eight patients with chronic neck pain and restricted upper cervical rotation were randomized in three groups: Upper cervical translatoric mobilization group, inhibitory suboccipital technique group, or control group. The neck disability index, active cervical mobility, and the flexion–rotation test were assessed at baseline (T0), after the treatment (T1), and at three-month follow-up (T2). *Results*: There were no statistically significant differences between groups in neck disability index. The upper cervical translatoric mobilization group showed a significant increase in the flexion–rotation test to the more restricted side at T1 (F = 5.992; *p* < 0.004) and T2 (F = 5.212; *p* < 0.007) compared to the control group. The inhibitory suboccipital technique group showed a significant increase in the flexion–rotation test to the less restricted side at T1 (F = 3.590; *p* < 0.027). All groups presented high percentages of negative flexion–rotation tests. (T1: 69.2% upper neck translator mobilization group; 38.5% suboccipital inhibition technique group, 19.2% control group; at T2: 80.8%; 46.2% and 26.9% respectively). No significant differences in the active cervical mobility were found between groups. Conclusion: Adding manual therapy to a conventional physical therapy protocol for the upper cervical spine increased the flexion–rotation test in the short- and mid-term in patients with chronic neck pain. No changes were found in the neck disability index and the global active cervical range of motion.

## 1. Introduction

Neck pain is commonly defined as “pain located between the occiput and the third thoracic vertebra” [1]. Mechanical neck pain has a yearly incidence of 12/1000 patients, and is among the most common reasons for visiting a primary care physician [2]. Neck pain often becomes chronic when lasting longer than 3 months [3] and generating sick-leave resulting in a high cost for the society [4,5].

- Patients with mechanical neck pain often experience reduction in neck mobility [6,7]. When reduction in neck mobility affects the upper cervical spine, a relevant reduction in the overall cervical range of motion may be expected. The cervical range of motion deficit could be especially present in the transverse plane, since around 60% of the cervical movement occurs in the upper cervical spine [8]. Several studies have analyzed the effect of myofascial [9] or articular [10,11,12,13] techniques to increase the reduced upper cervical spine-range of motion. These studies have shown an increase in the flexion–rotation test, a test measuring upper cervical spine range of motion in the transverse plane. In order to promote safety in the management of the upper cervical spine, the International Federation of Orthopedic Manipulative Physical Therapists (IFOMPT) recommends avoiding techniques which use maximal end range cervical rotation and extension [14].

Coulter ID et al. (2019) in a systematic review and meta-analysis on the topic of manipulation and mobilization for treating chronic nonspecific neck pain, concluded that studies published since January 2000 provide low-moderate quality evidence. Various types of manipulation and/or mobilization will reduce pain and improve function for chronic nonspecific neck pain compared to other interventions. It appears that multimodal approaches, in which multiple treatment approaches are integrated, might have the greatest potential impact [15]. However, none of the included articles focus on assessing and treating the upper cervical spine.

The upper cervical translatoric mobilization [16] and the inhibitory suboccipital technique [17] follow IFOMPT’s recommendations. Previous studies have shown that these techniques increase the range of motion in subjects with restricted upper cervical spine mobility [18], and in patients with cervicogenic headache and upper cervical spine dysfunction [13].

Recently, Rodriguez-Sanz J., et al. (2020) published an article using translatoric manual therapy techniques for the upper cervical spine combined with cervical exercises in patients with chronic neck pain and restricted upper cervical spine mobility [19]. However, these authors used a set of articular techniques and did not evaluate the effect of each of them in isolation or the effect of the inhibitory suboccipital technique.

Therefore, the effects of upper cervical translatoric mobilization or inhibitory suboccipital technique within a physical therapy approach in patients with chronic mechanical neck pain are unknown. 

The purpose of this study was to evaluate the effect of adding upper cervical translatoric mobilization and inhibitory suboccipital technique to a conventional physical therapy protocol in patients with chronic mechanical neck pain, restricted upper cervical rotation on disability, and cervical range of motion. 

The hypothesis was that adding upper cervical spine manual therapy to a conventional physical therapy protocol in patients with chronic mechanical neck pain and restricted upper cervical rotation would show improved effects on disability and cervical range of motion.

## 2. Material and Methods

### 2.1. Study Design

A simple blind (evaluator) randomized (1:1:1) controlled clinical trial was conducted. The study was registered at www.clinicaltrials.gov (NCT02832232). The Clinical Research Ethics Committee of the IDIAP Jordi Gol (reference number P16/068) approved the study and all patients provided written informed consent. The CONSORT statement was followed in this study.

### 2.2. Subjects and Sample Size

Patients with chronic mechanical neck pain were recruited from a Primary Care Center of Servei de Rehabilitació Baix Llobregat Centre, Cornella de Llobregat, Barcelona (Spain), between July 2016 and October 2018. 

The sample size was calculated using the GRANMO 7.12 program based on the statistics of Izquierdo-Pérez et al. (2014) [20]. The main variable used for the sample size calculation in our study was the neck disability index questionnaire [20]. The necessary statistics and the minimum differences to be detected between the groups were determined according to this previously mentioned study. We used an α risk of 0.05, a two-sided test, and a β risk of 0.20. For the neck disability index variable, we used an estimated common standard deviation of 6.8 [20] and a minimum expected difference of 5.8 [20]. We estimated a follow-up loss of 15%, which would require 26 subjects per group and a total sample of at least 78 subjects.

The inclusion criteria were patients over 18 years-old, with a clinical diagnosis of chronic mechanical neck pain, and a positive flexion–rotation test (asymmetry of >10° between sides or less than 32° in any direction) [21,22]. The exclusion criteria were any contraindication to receive manual therapy [14], presence of pacemaker, inability to perform the flexion–rotation test or lie in supine, and involvement in pending litigation.

### 2.3. Randomization and Allocation

Patients were simply randomly assigned to the Upper Neck Translatoric Mobilization, the Suboccipital Inhibitory Technique, or the Control group. For the randomization process, an external evaluator generated a random assignment list before the recruitment of the patients with a computer program (www.random.org) that generated a list of sequential numbers (from 1 to 78). Each one of them was assigned to one of the three study groups and the treatment was applied according to the group assigned to the subject’s number by the computer program. Assignments were placed in a concealed opaque envelope. Evaluator was blinded to the group assignment.

## 3. Measurements

- The primary outcome measures reported were neck disability and cervical mobility. Active cervical range of motion was measured in all planes for global cervical mobility and in the sagittal plane for upper cervical spine. Flexion–rotation test was measured to assess passive upper cervical spine range of motion in the transverse plane. Demographic variables were also registered (Table 1).

The neck disability index is a self-administered questionnaire with 10 sections, each with 6 possible answers representing 6 progressive levels of functional disability, 0 being the lowest level and 5 being the highest level in each section. Total scores ranged from 0 to 50 points, with higher scores indicating greater disability (0–4 = no disability; 5–14 = mild; 15–24 = moderate; 25–34 = severe and above 34 = complete). This questionnaire has an excellent test-retest reliability (ICC 0.97) and its translation to Spanish was validated [22,23]. The score for minimal detectable change is 5 points out of 50. To achieve a minimum clinically relevant difference, a reduction of 7 points out of 50 is recommended [24].

- For the active mobility testing, patients were asked to sit upright and move their head as far as they could without pain [25]. For the passive upper cervical spine mobility testing, a flexion–rotation test was performed according to Hall et al. [21]. The direction of the flexion–rotation test with less movement was considered the “flexion–rotation test to the more restricted side” and vice versa. The CROM device (Plastimo Airguide, Buffalo Groove, IL, USA) is a reliable and valid method for measuring active and passive cervical mobility [26]. Two measurements of each movement were performed, and the mean value was used for further analysis. The minimal detectable change in active range of motion is between 5°–10° [27,28] and in flexion–rotation between 4.7°–7° [29] using the CROM device.
- Measurements were performed at baseline (T0), at the end of the treatment (T1), and at three-months follow-up (T2). A researcher with 17 years of experience and specifically trained performed the measurements. This researcher was blinded to the allocation group of each patient.

## 4. Intervention

For three weeks, all groups received fifteen sessions of a conventional treatment based on superficial thermotherapy, cervical stretching and self-traction, thoracic mobilization, and pain education (advice to activate, advice on stress-coping skills, workplace ergonomics, and self-care strategies with an educational talk). 

This treatment consisted of autostretching exercises for the upper trapezius, elevator scapulae, and pectoralis major muscles. Stretching lasted 15 s and each exercise was repeated 10 times with a 5 s rest. 

Cervical spine auto-traction. Patients applied a cranial force with their hands placed under the occiput and mastoid process. Each mobilization lasted 15 s and each exercise was repeated 10 times with a 5 s rest. 

Thoracic spine auto-mobilization with the Kaltenborn’s wedge was performed. Patients performed a dorsal push with their body on the wedge at each dorsal segment. Each mobilization lasted 15 s and was repeated 2 times in each segment with a 5 s rest.

Additionally, the upper cervical translatoric mobilization and inhibitory suboccipital technique groups received 6 sessions consisting of 5 min of upper cervical spine translatoric mobilizations and 5 min of the pressure inhibition technique, respectively. A therapist with 25 years of clinical experience performed all of the interventions. 

**Figure 1:**
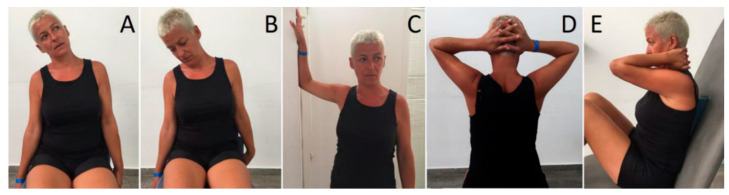
Conventional physical therapy treatment. (**A**) Stretching of the upper trapezius muscle; (**B**) stretching of the elevator scapula muscle; (**C**) stretching of the pectoralis major muscle; (**D**) cervical spine auto-traction; (**E**) thoracic spine auto-mobilization.

In the upper cervical translatoric mobilization group, patients were positioned supine with the upper cervical spine around the mid-position. The therapist placed a hand dorsally at the level of C1 with the radial border of the index finger and its metacarpophalangeal joint. The other hand was placed posteriorly under the occiput, with the shoulder contacting ventrally on the patient’s forehead. The mobilization force was directed dorsally from the shoulder to the patient’s forehead until feeling a marked resistance, and then slightly more pressure was applied to perform a stretching mobilization [13,30] (Figure 1).

**Figure 2:**
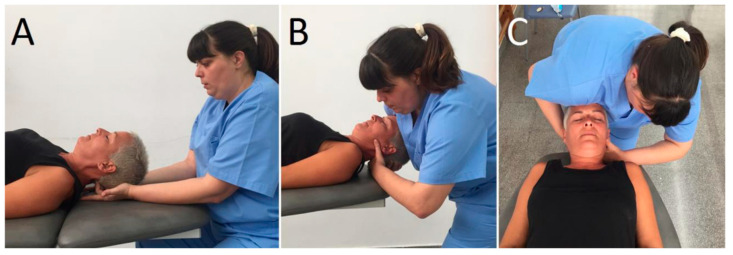
(**A**) Pressure inhibition technique application. (**B**,**C**) lateral and cranial view of the upper cervical translatoric mobilization (UCTM) application.

- In the inhibitory suboccipital technique, patients were positioned in supine. The therapist was sitting with their forearms resting on the head-end of the table, metacarpophalangeal joints of middle and ring finger flexed 90°, and applying ventral pressure in the suboccipital area with the patient’s head resting in the therapist’s hands [18]. The applied pressure was adjusted according to the therapist´s perception (Figure 2).

Patients were recommended to continue with cervical stretching, self-traction, and thoracic mobilization when the intervention finished.

## 5. Statistical Analysis

The SPSS program version 20.0 was employed to perform statistical analysis. A descriptive analysis of the baseline characteristics of the sample was performed. The assumption of normality was assessed using the Kolmogorov–Smirnov test. The intention-to-treat principle was followed in the analysis of the results [31]. Missing observations were filled in with the latest data of the same patient (Last-Observation-Carried-Forward method). A 3 × 3 mixed model analysis of variance (ANOVA) was used to compare between-group changes over the three measurement periods for all continuous level data. For within-group changes over the measurement periods, repeated measures (ANOVA) with a Bonferroni post hoc test was used. For the neck disability index variable, Fisher´s exact test was used. Effect sizes were calculated using Cohen’s d coefficient for quantitative variables. An effect size >0.8 was considered large; around 0.5, intermediate; and <0.2, small. For qualitative variables, Cramer´s V was used to calculate the effect size. An effect size >0.5 was considered strong; between 0.5–0.3, intermediate; and <0.3, small effect [32]. A *p* value < 0.05 was considered significant.

## 6. Results

**Figure 3:**
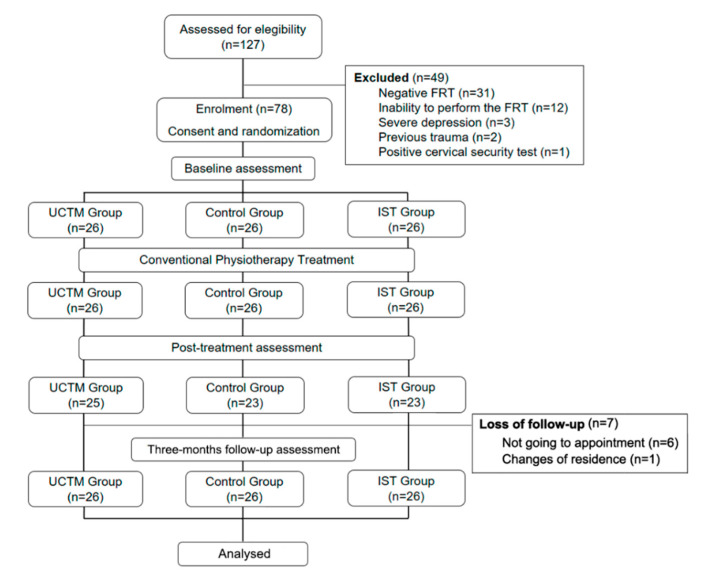
Flow chart.

Seventy-eight participants were recruited, 64 were women (82.1%) and 14 were men (17.9%) with a global mean age of 59.96 years (SD 13.30). Table 1 shows the baseline characteristics of the sample at baseline. Figure 3 shows the consort diagram of the study. 

## 7. Neck Disability Index

There were no statistically significant differences in the between-group analysis (Table 2).

## 8. Global Active Cervical Range of Motion

There were no statistically significant differences in the between-group analysis (Table 3). 

In the within-group analysis, a significant increase in active cervical range of motion was observed at T1 for the inhibitory suboccipital technique group in right (F = 6.419; *p* < 0.022) and left side-bending (F = 7.006; *p* < 0.03), and for the upper cervical translatoric mobilization group in left side-bending (F = 11.487; *p* < 0.006). At T2, the inhibitory suboccipital technique group showed a statistically significant increase in extension (F = 4.018; *p* < 0.023), right side-bending (F = 6.419; *p* < 0.002), and left rotation (F = 4.706; *p* < 0.044) in the within-group analysis (Table 4). 

## 9. Upper Cervical Range of Motion

In the between-group analysis (Table 3), both upper cervical translatoric mobilization (F = 984.174; *p* < 0.004) and inhibitory suboccipital technique (F = 615.298; *p* < 0.027) groups showed a statistically significant increase in the flexion–rotation test to the more restricted side compared to the Control Group at T1. At T2, only the upper cervical translatoric mobilization group maintained the improvement (*p* < 0.007). 

A significant increase in flexion–rotation test was observed at T1 for all groups (*p* < 0.05) (Table 4) in the within-group analysis. The Control Group experienced significant increase in the flexion–rotation test to the more restricted side (F = 6.977; *p* < 0.021), and a significant decrease in flexion–rotation test to the less restricted side (F = 7.138; *p* < 0.002). The inhibitory suboccipital technique group showed a statistically significant increase in the flexion–rotation test to the more restricted side (F = 16.518; *p* < 0.001). The upper cervical translatoric mobilization group also showed a significant increase in the flexion–rotation test to the more restricted side (F = 59.194; *p* < 0.001). The increase in flexion–rotation test to the more restricted side was maintained at T2 in all groups (control (*p* < 0.017), Inhibitory suboccipital technique (*p* < 0.044), and upper cervical translatoric mobilization (*p* < 0.001)). The reduction of the flexion–rotation test to the less restricted side compared to baseline was also maintained at T2 for the control group (F = 7.138; *p* < 0.047).

All groups showed a significant percentage of subjects who presented a negative flexion–rotation test. At T1, 69.2% of the upper neck translator mobilization group, 38.5% of the suboccipital inhibition technique group, and 19.2% of the control group. At T2 80.8%, 46.2%, and 26.9% respectively. 

There were no harms or adverse events reported in this study.

## 10. Discussion

The present study has found that adding both upper cervical manual therapy techniques to a protocol of physical therapy showed statistically significant differences in the flexion–rotation test range of motion. However, this improvement in upper cervical range of motion in the transverse plane compared to the control group was not associated to improvements in neck disability and global active cervical range of motion. 

In the present study, we found an improvement in all groups in neck disability with no differences between groups. These results are comparable to those of previous studies using multimodal approaches including manual therapy, cervical exercises, and other physical therapy techniques [33]. The results are superior to studies where regional mobilization or manipulation in isolation were performed [1,20,34]. These results strengthen the use of multimodal treatment therapies in the primary care approach for chronic neck pain.

Although there were not statistical differences among the groups, the inhibitory suboccipital technique resulted in a larger increase in active cervical range of motion at T1 and T2. It has been hypothesized that trigger points could be responsible for restricted range of motion [35,36]. The pressure applied on the suboccipital muscles could produce neurophysiological effects, diminishing cervical muscles tightness [37], and increasing active cervical range of motion. Some studies support the effect of muscle compression in the active cervical range of motion increase [38,39]. However, the active cervical range of motion increase in upper cervical translatoric mobilization and inhibitory suboccipital technique groups was inferior to the minimal detectable difference of the CROM device (5°–10°) [26,27]. It is possible that multisegmental structures like myofascial tissues limit active cervical range of motion testing and upper cervical spine range of motion improvement did not translate into a global cervical range of motion improvement.

The active cervical range of motion increase in our study was lower than other studies where more specific techniques were applied to improve dysfunctions in the lower cervical spine [20,33,40]. Larger range of motion increase has been obtained in all planes with multimodal approaches and the application of techniques on restricted lower cervical segments in patients with chronic cervicalgia [20]. The different methodology and characteristics of the sample among studies could explain these differences. In those studies, the restricted mobility was present in the lower cervical spine, the techniques were applied on a restricted lower cervical vertebra and the sample age was lower.

According to the diagnostic criteria for a positive flexion–rotation test [21,22], all groups showed a significant increase in this test. The groups adding manual therapy for the upper cervical spine showed the highest percentages of negative flexion–rotation test at T1 and T2.

Only upper cervical translatoric mobilization and inhibitory suboccipital technique groups overcame the minimal detectable change in the flexion–rotation test [11]. The increase of our Control group is comparable to the increase of 5° in the placebo group reported by Hall et al. (2009) [21]. At T1, only the upper cervical translatoric mobilization group obtained a significant increase in flexion–rotation test range of motion compared to the Control group. This result is similar to other studies in patients with positive flexion–rotation test [12,19] and with cervicogenic headache [13]. At T2, all groups obtained a significant increase in the restricted side of the flexion–rotation test, but only groups with an upper cervical spine technique resulted in an increase exceeding the minimal detectable change. Only the upper cervical translatoric mobilization group showed a significant flexion–rotation test increase with a large effect size compared to Control group. The results are comparable or superior to studies applying manual techniques at the rotation end-range in asymptomatic subjects [10], and patients with neck pain [41] or cervicogenic headache [11]. Upper cervical translatoric mobilization could be considered as safer in the upper cervical spine approach avoiding end-range positions and meeting the international recommendations [14]. Malo et al. (2017) [13] applied upper cervical translatoric mobilization alone in patients with cervicogenic headaches and reported an increase in upper cervical spine-range of motion slightly inferior to the present study. Probably, our combination of multimodal treatment and upper cervical translatoric mobilization could explain this difference. upper cervical translatoric mobilization is intended to improve upper cervical rotation [42]. Clinically, this technique could be applied when improving upper cervical rotation restriction due to the specific improvement shown in our study. However, this technique should be associated to a multimodal approach for a chronic neck pain sample.

## 11. Limitations

Firstly, the results are limited to a sample presenting several inclusion and exclusion criteria. Only one therapist provided the treatment which may limit the generalization of the results. Secondly, the implementation of the treatment was limited in time to maintain the functioning of the Primary Care Service. Therefore, neither the dosage nor the most appropriate upper cervical spine technique was adapted to the individual patients’ clinical presentation. Thirdly, due to differences in sample characteristics, methodology, or treatment procedures, the comparison among the studies is difficult. Lastly, only short and mid-term follow up were analyzed in the present study. A long-term randomized trial is needed to determine the effect sustained over-time.

## 12. Conclusions

Adding upper cervical translatoric mobilization to a conventional physical therapy protocol increased upper cervical spine range of motion both in the short- and mid-term in patients with chronic mechanical neck pain and restricted upper cervical rotation. Adding inhibitory suboccipital technique to a conventional physical therapy protocol improved the flexion–rotation test in the short-term. None of the added techniques had better results in the neck disability index and the global active cervical range of motion compared to the conventional physical therapy protocol.

## Figures and Tables

**Table 1:** Descriptive statistics at baseline.

Variables	IST Group (*n* = 26)	UCTM Group (*n* = 26)	Control Group (*n* = 26)
Age in years (mean ± SD)	59.31 ± 12.41	58.92 ± 11.75	61.65 ± 15.77
Sex (*n* and %)			
Man	5 (19.2%)	5 (19.2%)	4 (15.4%)
Woman	21 (80.8%)	21 (80.8%)	22 (84.6%)
Work activity (*n* and %)			
Active	12 (46.2%)	11 (42.3%)	9 (34.6%)
Not working	14 (53.8%)	15 (57.7%)	17 (65.4%)
Work with manual loads (*n* and %)			
Yes	7 (26.9%)	8 (30.8%)	3 (11.5%)
No	17 (65.4%)	13 (50%)	21 (80.8%)
Varied	2 (7.7%)	5 (19.2%)	2 (7.7%)
Analgesic medication (*n* and %)			
Yes	16 (61.5%)	10 (38.5%)	15 (57.7%)
No	10 (38.5%)	16 (61.5%)	11 (42.3%)
FRT Restricted Side (*n* and %)			
Right	15 (57.7%)	19 (73.1)	18 (69.2%)
Left	11 (42.3%)	7 (26.9)	8 (30.8%)
Duration of symptoms in months (mean ± SD)	25.73 ± 25.21	24.88 ± 23.78	19.81 ± 19.25
Global Cervical Spine ROM (°) (mean ± SD)			
Flexion	55.50 ± 12.04	60.29 ± 14.62	59.42 ± 9.67
Extension	34.86 ± 8.46	40.44 ± 8.01	39.56 ± 9.63
Lateral Flexion (Right)	26.38 ± 6.65	29.33 ± 9.97	26.65 ± 6.77
Lateral Flexion (Left)	26.31 ± 7.88	30.19 ± 8.62	28.56 ± 7.80
Rotation (Right)	45.56 ± 8.54	50.48 ± 11.46	49.83 ± 8.08
Rotation (Left)	46.19 ± 12.78	53.48 ± 12.79	50.83 ± 12.55
Upper Cervical Spine ROM (°) (mean ± SD)			
Flexion	10.54 ± 5.28	11.94 ± 4.58	12.42 ± 5.60
Extension	9.96 ± 4.36	10.35 ± 4.38	11.61 ± 4.28
FRT More restricted	23.92 ± 6.44	25.52 ± 7.65	25.35 ± 6.38
FRT Less restricted	36.04 ± 7.28	38.23 ± 6.14	37.19 ± 7.04
Neck Disability Index (*n* and %)			
No Disability	3 (11.5%)	2 (7.7%)	1 (3.8%)
Mild Disability	10 (28.5%)	14 (53.8%)	17 (65.4%)
Moderate Disability	12 (46.2%)	8 (30.8%)	7 (26.9%)
Severe Disability	1 (3.8%)	1 (3.8%)	1 (3.8%)
Complete Disability	0 (0%)	1 (3.8%)	0 (0%)

Abbreviations: IST, inhibitory suboccipital technique; UCTM, upper cervical translatoric mobilization; FRT, flexion–rotation test; ROM, range of motion.

**Table 2:** Outcomes Neck Disability Index values.

		T0	T1		T2	
		Baseline	End of Treatment		3 Months Post-Treatment	
		*n* (%)	*n* (%)	*p* and r Value	n (%)	*p* and r Value
No Disability	Control	1 (3.8%)	6 (23.1%)	*p* < 0.122 r = 0.23	9 (34.6%)	*p* < 0.063 r = 0.24
	IST	3 (11.5%)	7 (26.9%)		7 (26.9%)	
	UCTM	2 (7.7%)	12 (46.2%)		15 (57.7%)	
Mild Disability	Control	17 (65.4%)	14 (53.8%)		13 (50%)	
	IST	10 (28.5%)	15 (57.7%)		17 (65.4%)	
	UCTM	14 (53.8%)	13 (50%)		11 (42.3%)	
Moderate Disability	Control	7 (26.9%)	5 (19.2%)		4 (15.4%)	
	IST	12 (46.2%)	4 (15.4%)		2 (7.7%)	
	UCTM	8 (30.8%)	1 (3.8%)		0 (0%)	
Severe Disability	Control	1 (3.8%)	1 (3.8%)		0 (0%)	
	IST	1 (3.8%)	0 (0%)		0 (0%)	
	UCTM	1 (3.8%)	0 (0%)		0 (0%)	
Complete Disability	Control	0 (0%)	0 (0%)		0 (0%)	
	IST	0 (0%)	0 (0%)		0 (0%)	
	UCTM	1 (3.8%)	0 (0%)		0 (0%)	

Abbreviations: IST, inhibitory suboccipital technique; UCTM, upper cervical translatoric mobilization; r; Cramer’s V.

**Table 3:** Outcomes variable between-groups values.

		T1			T2		
		Mean (95% CI)	*p*-Value	ES	Mean (95% CI)	*p*-Value	ES
IST vs. UCTM	Flexion GCS	−4.44 (−12.20/3.31)	0.494	0.04	2.26 (−6.14/10368)	1.000	0.17
	Extension GCS	−3.96 (−10.67/2.74)	0.457	0.35	2.40 (−4.29/9.09)	1.000	0.27
	Right SB	−2.27 (−6.33/5.79)	1.000	0.03	1.52 (−4.37/7.41)	1.000	0.18
	Left SB	−1.03 (−6.38/4.31)	1.000	0.13	0.23 (−0.55/1.01)	1.000	0.17
	Right Rotation GCS	1.00 (−6.74/8.74)	1.000	0.08	−0.23 (−7.73/7.27)	1.000	0.02
	Left Rotation GCS	−1.08 (−8.92/6.77)	1.000	0.09	1.94 (−5.02/8.90)	1.000	0.21
	Flexion UCS	1.06 (−3.11/5.22)	0.874	0.17	−0.25 (−5.12/4.62)	1.000	0.03
	Extension UCS	−1.06 (−4.85/2.74)	1.000	0.20	0.33 (−3.55/4.20)	1.000	0.06
	FRT More restricted	−2.02 (−6.86/2.82)	0.931	0.30	−4.69 (−9.88/0.49)	0.089	0.66
	FRT Less restricted	3.02 (−2.32/8.36)	0.512	0.37	−2.44 (−8.59/3.70)	1.000	0.27
IST vs. Control	Flexion GCS	−1.48 (−9.23/6.27)	1.000	0.13	1.57 (−6.83/9.99)	1.000	0.13
	Extension GCS	1.90 (−4.81/8.61)	1.000	0.18	6.13 (−0.55/12.82)	0.083	0.60
	Right SB	2.15 (−3.90/8.21)	1.000	0.27	0.94 (−4.94/6.83)	1.000	0.12
	Left SB	3.50 (−1.85/8.85)	0.340	0.44	0.35 (−0.41/1.14)	0.762	0.28
	Right Rotation GCS	3.48 (−4.26/11.22)	0.824	0.29	3.54 (−3.96/11.04)	0.755	0.33
	Left Rotation GCS	−1.11 (−8.96/6.73)	1.000	0.10	4.56 (−2.40/11.52)	0.399	0.42
	Flexion UCS	1.81 (−2.36/5.97)	1.000	0.30	3.19 (−1.68/8.06)	0.338	0.41
	Extension UCS	1.75 (−2.05/5.56)	0.788	0.33	3.04 (−0.84/6.92)	0.176	0.53
	FRT More restricted	4.65 (−0.19/9.49)	0.064	0.60	1.96 (−3.22/7.14)	1.000	0.25
	FRT Less restricted	5.84 (0.50/11.19)	0.027	0.81	1.90 (−4.24/8.05)	1.000	0.19
UCTM vs. Control	Flexion GCS	2.96 (−4.79/10.71)	1.000	0.20	−0.69 (−9.10/7.72)	1.000	0.06
	Extension GCS	5.86 (−0.84/12.58)	0.107	0.19	3.73 (−2.95/10.42)	0.529	0.36
	Right SB	2.42 (−3.63/8.48)	0.992	0.25	−0.57 (−6.46/5.31)	1.000	0.06
	Left SB	4.54 (−0.81/9.89)	0.123	0.57	0.13 (−0.64/0.91)	1.000	0.27
	Right Rotation GCS	2.48 (−5.26/10.22)	1.000	0.25	3.77 (−3.73/11.27)	0.667	0.37
	Left Rotation GCS	-0.04 (−7.88/7.81)	1.000	0.00	2.61 (−4.34/9.57)	1.000	0.32
	Flexion UCS	0.75 (−3.41/4.91)	1.000	0.12	3.44 (−1.43/8.31)	0.263	0.53
	Extension UCS	2.80 (−0.99/6.60)	0.223	0.47	2.71 (−1.16/6.59)	0.273	0.47
	FRT More restricted	6.67 (1.83/11.51)	0.004	0.99	6.65 (1.47/11.84)	0.007	0.84
	FRT Less restricted	2.83 (−2.52/8.17)	0.597	0.34	4.34 (−1.80/10.49)	0.262	0.53

Abbreviations: IST, inhibitory suboccipital technique; UCTM, upper cervical translatoric mobilization; GCS, global cervical spine, UCS, upper cervical spine; SB, side-bending; FRT, flexion–rotation test; ES, effect size Cohen’s d.

**Table 4:** Outcomes variable within-group values.

		T0	T1					T2				
		Baseline	End of Treatment	Difference Between Baseline				3 Months Post-Treatment	Difference Between Baseline			
		Mean ± SD	Mean ± SD	Mean ± SD	(95% CI)	*p*-Value	ES	Mean ± SD	Mean ± SD	(95% CI)	*p*-Value	ES
Control Group	Flexion GCS	59.42 ± 9.67	58.96 ± 7.69	−0.46 ± 7.91	−4.44/−3.52	1.000	0.05	61.85 ± 8.96	2.42 ± 10.59	−2.91/7.75	0.764	0.26
	Extension GCS	39.56 ± 9.63	37.37 ± 8.72	−2.19 ± 10.20	−7.33/2.94	0.851	0.24	38.27 ± 10.30	−1.29 ± 11.69	−7.17/4.59	1.000	0.13
	Right SB	26.65 ± 6.77	28.29 ± 7.41	1.63 ± 8.92	−1.97/5.24	1.000	0.23	30.23 ± 7.65	3.58 ± 9.46	−0.24/7.40	0.196	0.50
	Left SB	28.56 ± 7.80	29.33 ± 8.48	0.77 ± 7.98	−3.24/4.78	1.000	0.09	28.56 ± 7.80	0.00 ± 0.00	0.00/0.00	1.000	0.00
	Right Rotation GCS	49.83 ± 8.08	50.31 ± 8.61	0.48 ± 9.56	−3.38/4.34	1.000	0.06	51.85 ± 8.05	2.02 ± 8.46	−1.40/5.43	0.704	0.02
	Left Rotation GCS	50.83 ± 12.55	51.94 ± 10.57	1.12 ± 11.04	−4.43/6.67	1.000	0.10	52.37 ± 13.01	1.54 ± 9.86	−3.42/6.49	1.000	0.12
	Flexion UCS	12.42 ± 5.60	12.56 ± 5.09	0.13 ± 6.22	−2.38/2.65	1.000	0.03	9.75 ± 3.31	−2.67 ± 7.14	−5.56/0.21	0.203	0.58
	Extension UCS	11.61 ± 4.28	11.79 ± 5.16	0.17 ± 5.99	−2.25/2.59	1.000	0.04	10.56 ± 4.13	−1.06 ± 5.89	−3.44/1.32	1.000	0.25
	FRT More Restricted	25.35 ± 6.38	29.79 ± 5.76	4.44 ± 7.71	0.56/8.32	0.021	0.73	31.77 ± 6.07	6.42 ± 8.56	2.11/10.74	0.017	1.03
	FRT Less restricted	37.19 ± 7.04	31.71 ± 4.64	−5.48 ± 7.32	−9.16/−1.80	0.002	0.92	32.63 ± 7.37	−4.56 8.97	−9.07/−0.04	0.047	0.63
IST Group	Flexion GCS	55.50 ± 12.04	53.56 ± 11.84	−1.94 ± 13.40	−7.36/3.47	1.000	0.16	59.50 ± 8.83	4.00 ± 13.47	−1.44/9.44	0.427	0.38
	Extension GCS	34.86 ± 8.46	34.58 ± 11.49	−0.29 ± 10.66	−5.65/5.08	1.000	0.03	39.71 ± 10.36	4.85 ± 8.51	0.56/9.13	0.023	0.51
	Right SB	26.38 ± 6.65	30.17 ± 8.10	3.79 ± 6.60	0.47/7.11	0.022	0.51	30.90 ± 8.59	4.52 ± 5.99	1.50/7.53	0.002	0.54
	Left SB	26.31 ± 7.88	30.58 ± 8.21	4.27 ±7.80	0.34/8.19	0.030	0.53	26.67 ± 8.63	0.37 ± 1.86	−0.57/1.30	0.981	0.04
	Right Rotation GCS	45.56 ± 8.54	49.52 ± 13.00	3.96 ± 14.16	−1.76/9.68	0.498	0.36	51.12 ± 11.82	5.56 ± 12.55	0.49/10.63	0.099	0.54
	Left Rotation GCS	46.19 ± 12.78	46.19 ± 12.50	0.00 ± 10.25	−5.16/5.16	1.000	0.00	52.29 ± 11.24	6.10 ± 11.85	0.13/12.06	0.044	0.51
	Flexion UCS	10.54 ± 5.28	12.48 ± 6.20	1.94 ± 5.84	−0.42/4.30	0.307	0.34	11.06 ± 7.25	0.52 ± 8.30	−2.83/3.87	1.000	0.08
	Extension UCS	9.96 ± 4.36	11.88 ± 4.44	1.92 ± 4.66	−0.04/3.81	0.137	0.44	11.94 ± 5.13	1.98 ± 5.66	−0.31/4.27	0.270	0.42
	FRT More Restricted	23.92 ± 6.44	33.02 ± 8.60	9.10 ± 7.82	−13.03/−5.16	0.001	1.20	32.31 ± 7.32	8.38 ± 7.01	4.86/11.91	0.001	1.22
	FRT Less restricted	36.04 ± 7.28	36.40 ± 7.92	0.37 ± 7.03	−3.17/3.90	1.000	0.05	33.38 ± 10.54	−2.65 ± 10.53	−7.95/2.64	0.631	0.29
UCTM Group	Flexion GCS	60.29 ± 14.62	62.79 ± 12.68	2.50 ±12.20	−2.43/7.43	0.918	0.18	62.02 ± 11.61	1.73 ± 12.90	−3.48/6.94	1.000	0.13
	Extension GCS	40.44 ± 8.01	44.12 ± 9.72	3.67 ± 8.66	−0.69/8.03	0.121	0.41	42.88 ± 6.28	2.44 ± 9.06	−2.12/7.00	0.545	0.34
	Right SB	29.33 ± 9.97	33.38 ± 8.07	4.06 ± 10.75	−0.28/8.40	0.197	0.45	32.33 ± 9.35	3.00 ± 10.01	−2.03/8.03	0.417	0.31
	Left SB	30.19 ± 8.62	35.50 ± 10.59	5.31 ± 7.84	1.36/9.25	0.006	0.55	30.33 ± 8.81	0.13 ± 0.69	−0.21/0.48	0.981	0.02
	Right Rotation GCS	50.48 ± 11.46	53.44 ± 11.63	2.96 ± 9.90	−1.04/6.96	0.420	0.26	56.27 ± 9.82	5.79 ± 11.71	1.06/10.52	0.055	0.54
	Left Rotation GCS	53.48 ± 12.79	54.56 ± 14.63	1.08 ± 13.18	−5.53/7.07	1.000	0.08	57.63 ± 10.93	4.15 ± 8.79	−0.27/8.58	0.071	0.32
	Flexion UCS	11.94 ± 4.58	12.83 ± 4.27	0.88 ± 6.32	−1.67/3.44	1.000	0.20	12.71 ± 3.95	0.77 ± 5.88	−1.60/3.14	1.000	0.18
	Extension UCS	10.35 ± 4.38	13.33 ± 5.02	2.98 ± 6.02	0.55/5.41	0.055	0.63	12.00 ± 4.00	1.65 ± 5.57	−0.59/3.90	0.427	0.39
	FRT More Restricted	25.52 ± 7.65	36.63 ± 6.54	11.12 ± 5.65	8.27/13.95	0.001	1.56	38.60 ± 5.19	13.08 ± 7.25	9.42/10.73	0.001	2.00
	FRT Less restricted	38.23 ± 6.14	35.58 ± 7.32	−2.65 ± 9.09	−7.23/1.92	0.448	0.39	38.02 ± 5.53	−0.21 ± 7.37	−3.92/3.50	1.000	0.04

Abbreviations: SD, standard deviation; GCS, global cervical spine, UCS, upper cervical spine; SB, side-bending; FRT, Flexion–rotation test; *p*-value: ANOVA with repeated measures and Bonferroni post hoc test. ES, effect size Cohen’s d.
